# Effect of breathing conditions on relationships between impairment, breathing laterality and coordination symmetry in elite para swimmers

**DOI:** 10.1038/s41598-024-56872-y

**Published:** 2024-03-18

**Authors:** Ludovic Seifert, Adrien Létocart, Brice Guignard, Mohamed Amin Regaieg

**Affiliations:** 1 CETAPS UR3832, Faculty of Sport Sciences, University of Rouen Normandy, Boulevard Siegfried, Bâtiment 36A, 76130 Mont Saint Aignan, France; 2 Institut Universitaire de France (IUF), Paris, France; 3 LIBM UR7424 - Laboratoire Interuniversitaire de Biologie de la Motricité, Université Claude Bernard Lyon 1, Villeurbanne, France

**Keywords:** Motor control, Biomechanics, Kinematics, Para swimming, Expertise, Asymmetry, Motor control, Human behaviour

## Abstract

The aim was to investigate the effect of breathing conditions and swimming pace on the relationships between the impairment, the breathing laterality and motor coordination symmetry in elite front crawl Para swimmers. Fifteen elite Para swimmers with unilateral physical impairment or with visual impairment and unilateral breathing preference performed eight 25 m using four breathing conditions (every three strokes, every two strokes on preferred and non-preferred breathing side and apnea) at slow and fast paces in a randomized order. Multicamera video system and five sensors have been used to assess arm and leg stroke phases and to compute symmetry of arm coordination (SI_IdC_) and of leg kick rate (SI_KR_). Our findings emphasized motor coordination asymmetry whatever the breathing conditions and swimming paces, highlighting the influence of impairment. Multinomial logistic regression exhibited a high probability for motor coordination asymmetry (SI_IdC_ and SI_KR_) to be present in categories of Para swimmers with impairment and breathing laterality on the same side, suggesting the joined effect of unilateral impairment and unilateral breathing. Moreover, unilateral physical impairment and breathing laterality could also occur on different sides and generate motor coordination asymmetry on different sides and different levels (arms vs. legs). Finally, visual impairment seems amplify the effect of unilateral breathing on motor coordination asymmetry.

## Introduction

Asymmetries, both at kinematical and kinetical levels, are often considered as normal in unilateral human activities and sports (e.g., tennis, javelin throw), but they may affect performance and efficiency in cyclic and continuous activities such as pedalling, walking, swimming. In swimming, asymmetries could relate to several factors such as handedness (arm dominance), breathing laterality (in the case of unilateral breathing pattern), breathing action (i.e. a stroke during which the swimmer breathes vs does not breath)^1^.

Regarding the effect of breathing action, Formosa et al.^2^ observed an asymmetrical instantaneous net drag force stroke profile in both the breathing and non-breathing conditions of their test. However, within the breathing condition, expert swimmers compared to the less expert swimmers highlighted a lesser percentage of overlap between stroke phases on their breathing side, whereas a reduction in the percentage of overlap between stroke phases occurred on their breathing side in the non-breathing condition^2^. Psycharakis et al.^3^ did not observe any effect of breathing action on force production during tethered swimming despite the longer stroke cycle times (from one water hand entry to the subsequent one) when breathing. These findings at kinetical level resonated with those of Seifert et al.^4^ who observed that breathing action significantly amplified the asymmetric coordination on the preferential breathing side during a 100 m front crawl race simulation in non-expert swimmers in comparison to elite swimmers. In particular, a catch-up pattern of coordination (i.e., lag time between propulsive actions of the two arms) mainly occurred when breathing, while a superposition pattern of coordination (i.e. overlap of the propulsive actions of the upper limbs) occurred to the non-breathing side^4^.These results were confirmed by Cohen et al.^5^, who observed shorter propulsive phases (due to faster hand speed) on the breathing side than on the non-breathing side.

Some other research investigated the relationships between breathing laterality and asymmetry of motor organisation, without clear findings. For instance, when the peak force, the mean force, the impulse and the rate of force development have been investigated during tethered swimming test, a significant asymmetry between right and left sides have been observed without any effect of unilateral versus bilateral breathing preference^6^. Conversely, although symmetrical force production was observed in the majority of the swimmers (66.7%), when force asymmetry was observed, this asymmetry occurred to the non-preferred breathing side for this sample of high-level male swimmers^7^. Moreover, Seifert et al.^4^ observed that in expert and non-expert swimmers having unilateral breathing patterns (i.e. breathing every two strokes), an asymmetric coordination mainly related to breathing laterality. On the non-preferred breathing side, the arm probably controls and supports inhalation with the arm extended forward, whereas on the preferred breathing side the arm is responsible for the swimming rhythm and generates higher forces (e.g. the push phase occurs during exhalation on the same side), enabling the swimmer to associate propulsion with unilateral breathing^4^. In the same vein, when measuring the contribution of hand drag forces to the streamwise thrust, Cohen et al.^5^ also showed asymmetry between the left and right hands. In particular, when right unilateral breathing preference, the left hand showed two peaks in this drag force, one during the pull and one during the push with a dramatic drop off in force in between, whereas the right hand displayed only a single peak in the drag force during the propulsive phase^5^. To further explore the effect of breathing laterality, Seifert et al.^8^ compared breathing conditions which were hypothesised to lead either to symmetric or to asymmetric arm coordination. Their findings confirmed that bilateral breathing, apnea and breathing in a frontal snorkel led to symmetric arm coordination whereas unilateral breathing to the preferred and non-preferred sides led to asymmetric arm coordination^8^. From this state of art, arm coordination asymmetry would relate both to breathing action and to breathing laterality.

However, when the action of breathing is explored in swimmers with unilateral physical impairment (e.g. amputation or agenesis), one can wonder whether those swimmers should breath on the same side or on the opposite side of their impairment? Indeed, unilateral arm amputee swimmers exhibited larger shoulder roll amplitude^9^ and asymmetric coordination towards the affected than the unaffected side^10^, suggesting that breathing (action and laterality) could further increase those asymmetries. Recently, Santos et al.^11^ showed that impaired swimmers from S5 to S10 classes (https://www.paralympic.org/swimming/classification) had asymmetry in anteroposterior and mediolateral amplitudes of the stroke, arm coordination, duration of the recovery phase, in the hand speed during the downsweep phase, but also in the vertical amplitude of the upper limbs stroke as well as in the insweep and upsweep speeds. Santos et al.^11^ suggested that these asymmetries may relate to unilateral breathing, force imbalance between pairs of homologous muscles and motor control deficit; however, these relationships have been examined only by one research. Seifert et al.^12^ investigated the relationships between impairment, breathing and motor coordination symmetry and found that unilateral physical impairment was associated to asymmetric arm coordination (~ 83% of time, mostly at fast speeds), which always occurred to the side of the affected limb and was also associated to the preferential breathing side ~ 53% of time.

Beyond considering that unilateral breathing preference (i.e. breathing laterality) might amplify the potential motor coordination asymmetry associated to unilateral physical impairment, one can wonder whether *unilateral breathing* preference associated to visual impairment could also lead to motor coordination asymmetry. Indeed, a Delphi study showed that one most frequent race component that would be affected by visual impairment is the navigation perfectly in the middle of the lane^13^, which emphasized possible underlying relationships between the role of vision and motor asymmetries in order to control straight navigation in the lane. When comparing Para swimmers with higher to lower visual impairment (i.e. from S11 to S13), Malone et al.^14^ and Souto et al.^15^ observed lower clean swimming speed in S11 Para swimmers during 50-m, 100-m and 400-m freestyle race. Based on measurements of visual acuity and swimming performance, Fortin-Guichard et al.^16^ confirmed differences between functionally blind and partially sighted Para swimmers. Moreover, Malone et al.^14^ suggested that visual impairment might also affect the body roll refinement and breathing technique, but it has not been tested yet. Thus, in our current study, swimmers with visual impairment and unilateral breathing pattern were also accepted as they might exhibit motor control deficit in body roll refinement, motor coordination and swimming direction due to head rotation in the case of unilateral breathing.

To better understand whether unilateral breathing (action and laterality) could amplify motor coordination asymmetry associated to physical or visual impairment, our study built on previous research conducted on able-bodied expert swimmers^8^ to investigate the effect of breathing conditions, which would generate either symmetry or asymmetry of motor coordination in front crawl elite Para swimmers. We hypothesized that whatever the breathing conditions the Para swimmers (both with unilateral physical impairment or visual impairment) exhibited further motor coordination asymmetry than symmetry, which would mainly occur on the impairment side and/or on the preferential breathing side.

The second aim of our study was to examine the additional effect of swimming pace on this motor coordination asymmetry. Vila Dieguez and Barden^17^ showed an asymmetric body roll due to breathing (i.e. greater body roll on breathing side than on non-breathing side), and a decrease of body roll on both sides when swimming speed increased, which is concomitant with an increase of stroke rate^18^. Due to the lower time to breath (because of high stroke rate), we hypothesized that breathing (regardless the breathing conditions) would disrupt motor organisation by involving coordination asymmetry at fast swimming pace. Conversely, at slow swimming pace, the lower stroke rate might let more time to Para swimmers to synchronise inhalation with arm stroke phase organisation; thus, motor coordination might be less disturbed by the breathing conditions and consequently should be more symmetric.

## Results

### Effect of breathing conditions and swimming paces on stroking parameters and motor coordination

The two-way repeated measures ANOVA did not show any significant differences of the breathing conditions nor any significant interaction between the breathing conditions and the swimming paces on the stroking parameters and motor coordination (Table 1). On the average of the four breathing conditions, the two-way repeated measures ANOVA showed significant higher speeds (1.48 ± 0.17 m s^−1^) at fast pace than at slow pace (1.16 ± 0.13 m s^−1^) (*F*_1,13_ = 38.11,* p* < 0.001, *η*_P_^2^ = 0.746), which correspond to an increase of 78% between the slow and fast pace (knowing that a minimum of 40% was expected). The two-way repeated measures ANOVA also showed significant higher stroke rate (*F*_1,13_ = 93.39,* p* < 0.001, *η*_P_^2^ = 0.878), higher stroke length (*F*_1,13_ = 37.05,* p* < 0.001, *η*_P_^2^ = 0.74), higher index of coordination (IdC) (*F*_1,13_ = 11.63,* p* = 0.005, *η*_P_^2^ = 0.472) and higher leg kicking rate (KR) (*F*_1,13_ = 11.14,* p* = 0.007, *η*_P_^2^ = 0.503) at fast pace than at slow pace, but no significant effect of swimming paces on SI_IDC_ and SI_KR_. Taken together, the results of SI_IDC_ and SI_KR_ exhibited asymmetric arm coordination and asymmetric leg coordination regardless the breathing conditions and swimming paces, but with high standard deviation (Table 1).

**Table 1:** Mean and standard deviation (*SD*) of the stroking parameters and motor coordination for the four breathing conditions and the two swimming paces.

Swim paces	Breathing conditions	Kicking rate (Hz)		Absolute SI KR (%)		Speed (m s^−1^)		Stroke rate (Hz)		Stroke length (m)		IdC (%)		Absolute SI IdC (%)	
		Mean	SD	Mean	SD	Mean	SD	Mean	SD	Mean	SD	Mean	SD	Mean	SD
Slow	3 T	1.25	0.41	48.47	69.90	1.14	0.14	0.47	0.03	2.44	0.38	− 12.4	4.1	19.1	20.7
	A	1.23	0.42	56.82	70.11	1.17	0.13	0.47	0.06	2.56	0.56	− 12.2	4.7	22.9	24.1
	NP	1.26	0.41	44.39	71.70	1.18	0.14	0.49	0.06	2.43	0.41	− 10.9	3.9	33.6	26.4
	P	1.32	0.42	40.55	72.36	1.15	0.12	0.49	0.04	2.37	0.34	− 11.1	3.8	25.8	16.7
Fast	3 T	1.99	0.47	46.53	73.75	1.46	0.21	0.80	0.08	1.84	0.32	− 1.9	4.6	30.5	18.2
	A	2.17	0.53	41.87	75.70	1.49	0.18	0.85	0.10	1.79	0.35	0.1	4.7	29.7	19.0
	NP	1.97	0.44	42.87	75.18	1.48	0.17	0.76	0.09	1.97	0.34	− 3.5	4.8	28.5	17.4
	P	2.08	0.57	38.97	72.98	1.49	0.16	0.83	0.06	1.80	0.20	− 1.0	3.8	31.4	17.7

3 T: breathing every 3 strokes, A: Apnea, NP: breathing every 2 strokes on the non-preferential side; P: breathing every 2 strokes on the preferential side.

### Relationships between impairment, breathing laterality and motor coordination symmetry

**Figure 1:**
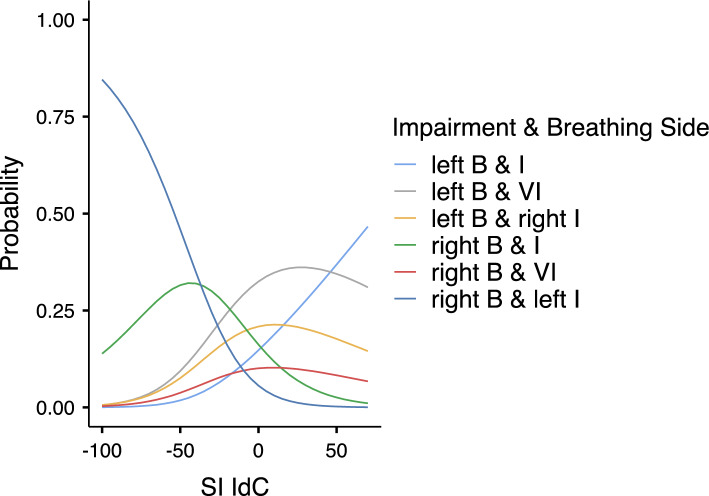
Probability of each category to occur predicated by estimated marginal means of SI_IdC_; B: breathing, I: Impairment.

**Figure 2:**
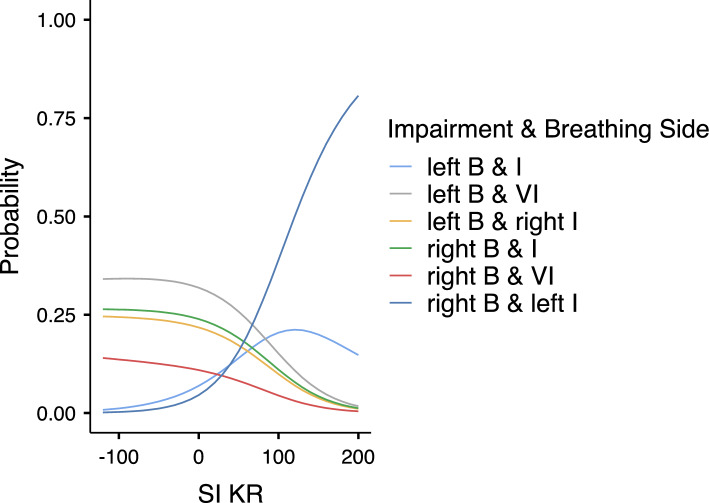
Probability of each category to occur predicated by estimated marginal means of SI_KR_; B: breathing, I: Impairment.

Then, multinomial logistic regression was performed based on SI_IdC_ and SI_KR_ predictors, as those predictors contributed significantly to predict the probability of categories to occur when each category was compared to the right physical impairment and right breathing laterality (used as a reference) (Table 2) (*X*^2^(5) = 39.0, *p* < 0.001 for SI_IdC_ and *X*^2^(5) = 38.6, *p* < 0.001 for SI_KR_). Considering AIC, *R*^2^_N_ and *X*^2^ values, a good model fitting occurred (AIC = 311, *R*^2^_N_ = 0.274, *X*^2^(10) = 80.1, *p* < 0.001). Then, the probability of each category to occur was predicted by the estimated marginal means of SI_IdC_ (Table 3 and Fig. 1) and by the estimated marginal means of SI_KR_ (Table 4 and Fig. 2) showing that the highest probability for the categories included swimmers with “*right breathing laterality and right impairment*” (Sw 1, 2, 10) and swimmers with “*right breathing laterality and left impairment*” (Sw 7, 8, 9) was predicted by SI_IdC_ < − 10% (i.e. right arm coordination asymmetry) and by SI_KR_ > 10% (i.e. left leg coordination asymmetry). Moreover, the highest probability for the category included swimmers with “*left breathing laterality and left impairment*” (Sw 5, 6) was predicted by SI_IdC_ > 10% (i.e. left arm coordination asymmetry) and by SI_KR_ > 10% (i.e. left leg coordination asymmetry). Finally, higher probability for the categories included swimmers with “*left breathing laterality and right impairment*” (Sw 3, 4), swimmers “*left breathing laterality and visual impairment*” (Sw 11, 12, 13) and swimmers “*right breathing laterality and visual impairment*” (Sw 14, 15) was predicated by either − 10% < SI_IdC_ < 10% (i.e. symmetric arm coordination) or SI_IdC_ > 10% (i.e. left arm coordination asymmetry) and either − 10% < SI_KR_ < 10% (i.e. symmetric leg coordination) or SI_KR_ < − 10% (i.e. right leg coordination asymmetry).

**Table 2:** Model coefficients for the prediction of each category (Unilateral Physical Impairment I, Visual Impairment VI & Breathing side B) to occur according to SI_IdC_ and SI_KR_ predictors.

Impairment & breathing side	Predictor	Estimate (Log odds ratio)	Estimate 95% CI		SE	Z	*p*	Odds ratio	Odds ratio 95% CI	
			Lower bound	Upper bound					Lower bound	Upper bound
Left B & I–right B & I	Intercept	− 0.661	− 1.721	0.399	0.541	− 1.221	0.222	0.516	0.178	1.491
	SI IdC	0.055	0.021	0.088	0.017	3.232	**0.001**	1.057	1.022	1.093
	SI KR	0.018	0.004	0.036	0.009	2.011	**0.044**	1.019	1.000	1.037
Left B & VI–right B & I	Intercept	0.686	− 0.101	1.474	0.402	1.708	0.088	1.987	0.903	4.370
	SI IdC	0.038	0.012	0.063	0.013	2.923	**0.003**	1.039	1.012	1.066
	SI KR	0.002	− 0.018	0.018	0.009	0.028	0.977	1.000	0.981	1.019
Left B & right I–Right B & I	Intercept	0.258	− 0.585	1.103	0.430	0.600	0.548	1.295	0.556	3.014
	SI IdC	0.033	0.006	0.060	0.013	2.412	**0.016**	1.034	1.006	1.063
	SI KR	− 0.001	− 0.020	0.020	0.010	− 0.017	0.986	1.000	0.979	1.020
Right B & VI–right B & I	Intercept	− 0.436	− 1.426	0.552	0.504	− 0.865	0.387	0.646	0.240	1.737
	SI IdC	0.033	0.001	0.066	0.016	1.960	0.050	1.034	1.000	1.068
	SI KR	− 0.001	− 0.026	0.024	0.013	− 0.095	0.924	0.999	0.973	1.025
Right B & left I–right B & I	Intercept	− 1.950	− 3.471	− 0.429	0.776	− 2.513	0.012	0.142	0.031	0.651
	SI IdC	− 0.028	− 0.061	0.004	0.016	− 1.706	0.088	0.972	0.940	1.004
	SI KR	0.029	0.009	0.048	0.009	2.975	**0.003**	1.030	1.010	1.049

Confidence Interval (CI), Standard Error (SE), Z-score and p-value.

Significant values are in bold.

**Table 3:** Probability of each category to occur predicated by estimated marginal means of SI_IdC_.

Estimated Marginal Means–SI IdC	Impairement & Breathing Side	Probability	SE	95% CI	
				Lower bound	Upper bound
Mean − 1SD = − 44.2	Right B & I	0.320	0.101	0.104	0.536
	Left B & I	0.025	0.020	− 0.018	0.069
	Left B & VI	0.119	0.054	0.003	0.235
	Left B & right I	0.093	0.049	− 0.011	0.199
	Right B & VI	0.046	0.034	− 0.027	0.120
	Right B & left I	0.394	0.103	0.173	0.615
Mean: − 10.5	Right B & I	0.214	0.062	0.080	0.348
	Left B & I	0.109	0.046	0.010	0.207
	Left B & VI	0.288	0.065	0.147	0.428
	Left B & right I	0.194	0.057	0.070	0.317
	Right B & VI	0.094	0.043	0.001	0.186
	Right B & left I	0.100	0.044	0.005	0.194
Mean + 1 SD = 23.2	Right B & I	0.074	0.038	− 0.008	0.157
	Left B & I	0.243	0.077	0.079	0.408
	Left B & VI	0.360	0.084	0.180	0.540
	Left B & right I	0.208	0.071	0.055	0.361
	Right B & VI	0.099	0.053	− 0.013	0.212
	Right B & left I	0.013	0.012	− 0.012	0.039

SI: symmetry index; IdC: Index of Coordination; SE: standard error; CI: confidence interval; SD: standard deviation.

**Table 4:** Probability of each category to occur predicated by estimated marginal means of SI_KR_.

Estimated marginal means—SI KR	Impairement & breathing side	Probability	SE	95% CI	
				Lower bound	Upper bound
Mean − 1SD = − 47.3	right B & I	0.257	0.099	0.045	0.468
	left B & I	0.030	0.018	− 0.009	0.071
	left B & VI	0.338	0.099	0.125	0.551
	left B & right I	0.236	0.090	0.043	0.428
	right B & VI	0.124	0.072	− 0.029	0.279
	right B & left I	0.012	0.010	− 0.009	0.034
Mean = 30.6	right B & I	0.214	0.062	0.080	0.348
	left B & I	0.109	0.046	0.010	0.207
	left B & VI	0.288	0.065	0.147	0.428
	left B & right I	0.194	0.057	0.070	0.317
	right B & VI	0.094	0.043	0.001	0.186
	right B & left I	0.100	0.044	0.005	0.194
Mean + 1SD = 108.4	right B & I	0.096	0.079	− 0.073	0.266
	left B & I	0.209	0.091	0.013	0.404
	left B & VI	0.132	0.083	− 0.044	0.310
	left B & right I	0.086	0.070	− 0.062	0.235
	right B & VI	0.038	0.047	− 0.061	0.139
	right B & left I	0.435	0.126	0.167	0.704

SI: symmetry index; KR: Kick Rate; SE: standard error; CI: confidence interval; SD: standard deviation.

## Discussion

Although able-bodied swimmers switched between symmetric and asymmetric arm coordination^8^ and body roll^19^ according to the requested breathing conditions (bilateral, frontal snorkel and apnea vs. unilateral), our findings confirmed our first hypothesis suggesting that whatever the breathing conditions, Para swimmers with unilateral physical impairment mainly exhibited motor coordination asymmetry rather than symmetry. In fact, Seifert et al.^8^ showed that in national able-bodied swimmers, breathing every two strokes on the preferential breathing side led to asymmetric arm coordination on this preferential breathing side, while breathing every two strokes on the non-preferential breathing side led to asymmetric arm coordination on this non-preferential breathing side, and finally, bilateral breathing and apnea led to symmetric arm coordination. According to Lerda and Cardelli^20^, it was suggested that breathing led to a longer relative duration of the entry and catch phase and a shorter relative duration of the pull phase on the breathing side than on the non-breathing side. Those findings emphasised the influence of breathing conditions on arm coordination asymmetry as national able-bodied swimmers adapted their motor coordination (i.e. arm coordination symmetry vs. asymmetry, and side of asymmetry) according to the breathing conditions. In our current study, arm coordination remained asymmetric regardless breathing conditions, which emphasized the influence of impairment. Based on the study of Lecrivain et al.^21^, which demonstrated that forearm amputee swimmer generated 40 to 70% force in comparison to an able-bodied swimmer, it is reasonable to consider that the unaffected side played an important role to propel and to compensate the asymmetry linked to unilateral physical impairment. In 10 × 25 m test incremented in speed, Seifert et al.^12^ showed that the physical impairment on one side was associated to arm coordination asymmetry on the same side for 83.6% of the trials. It could be hypothesised that the longer relative duration of the arm propulsive phase on the unaffected side (resulting in arm coordination asymmetry) would ensure the greatest part of the propulsion to compensate the lack of propulsion and balance encountered on the impaired side. This interpretation resonated with the findings of Gonjo et al.^9^ in unilateral arm amputee swimmers, showing a larger shoulder roll angle towards the amputee arm side, as shoulder roll asymmetry might be associated to arm coordination asymmetry. Therefore, the longer relative duration of the pull and/or push phases on the impaired side would explain the emergence of arm coordination asymmetry in order to maintain body balance and to ensure propulsion.

When physical impairment side and breathing side were considered together, the multinomial logistic regression exhibited a high probability for arm coordination asymmetry to be present in categories of Para swimmers with impairment and breathing laterality on the same side, suggesting the joined effect of unilateral impairment and unilateral breathing (laterality and action). Indeed, Para swimmers of “*left impairment & breathing laterality*” category (Sw 5, 6) exhibited left arm coordination asymmetry and leg coordination asymmetry, and Para swimmers of “*right impairment & breathing laterality*” category (Sw 1, 2, 10) exhibited right arm coordination asymmetry and left leg coordination asymmetry. In these two categories in which impairment and breathing laterality were on the same side, we hypothesised that on the breathing side, the arm was responsible for the swimming rhythm and generates higher forces (e.g. the push phase occurs during exhalation on the same side), enabling the swimmer to associate propulsion with unilateral breathing^4,5^ and to compensate the unilateral impairment, notably when this impairment was located at the lower-limb level (which was the case for four Para swimmers: Sw 1, 5, 6, 10). As unilateral arm amputation increased the shoulder angle roll on the affected side^9^ and as breathing also increased the body roll^17,19,22^, it appeared reasonable to suggest that when unilateral physical impairment and unilateral breathing occurred on the same side, they jointly generated arm coordination asymmetry.

Moreover, unilateral physical impairment and breathing laterality could also occur on different sides and being associated to motor coordination asymmetry. In particular, the multinomial logistic regression showed that “*left impairment & right breathing laterality*” category (Sw 7, 8, 9) was predicated by high probability of right arm coordination asymmetry and left leg coordination asymmetry. Similarly, the multinomial logistic regression also showed that “*right impairment & left breathing laterality*” category (Sw 3, 4) was predicated by high probability of left arm coordination asymmetry and right leg coordination asymmetry. Interestingly, the Para swimmers 7 and 9 had left impairment located at lower-limb level, which could explain their left leg coordination asymmetry, and breathed to the right side, which would explain their right arm coordination asymmetry. To sum up, it is interesting to note that both physical impairment and breathing laterality can generate motor coordination asymmetry on different sides and on different limbs (upper vs. lower). This interpretation fits a previous study^23^ that observed different ways of coordinating arms and legs in unilateral arm amputee swimmers.

Last, Para swimmers with visual impairment and unilateral breathing preference exhibited either symmetric or asymmetric motor coordination regardless breathing conditions. In particular, the multinomial logistic regression showed that “*visual impairment & left breathing laterality*” category (Sw 11, 12, 13) was predicated by high probability of left arm coordination asymmetry and by either symmetry or right leg coordination asymmetry, while “*visual impairment & right breathing laterality*” category (Sw 14, 15) was predicated by high probability of symmetric arm coordination and by either symmetric or right leg coordination asymmetry. Interestingly, the swimmers with visual impairment and left breathing preference (Sw11, Sw12, Sw13) corresponded to a category of our sample with the second highest probability to exhibit left arm coordination asymmetry. Knowing that their breathing preference was to the left side, it could be hypothesized that unilateral breathing contributed to generate arm coordination asymmetry as those Para swimmers had less visual control on arms movement. Indeed, previous studies observed that turning head to breath generated higher body roll on the breathing side^17,19,22^ and could disturb the arm stroke organisation^8,22^. However, this interpretation could not be generalized to all swimmers with visual impairment because swimmers Sw14 and Sw15 with visual impairment and right breathing preference remained mainly with arm coordination symmetry.

Our second hypothesis, postulating a greater effect of breathing conditions on motor coordination asymmetry at fast pace rather than at slow pace, was not confirmed. One reason might be the small range between slow and fast speeds (range of 0.3 m s^−1^) which could explain the lower speed achieved in sprint (1.49 m s^−1^) by elite Para swimmers in comparison to elite able-bodied swimmers. Our speed values and range between slow (400 m pace) and fast (50 m pace) speeds were similar to those found in previous studies involving S8, S9 Para swimmers^10,23,24^. Although the Para swimmers of our study did not exhibit greater arm coordination asymmetry at fast speed, they switched between catch-up coordination pattern at slow speeds to superposition coordination pattern at fast speed. In comparison, elite able-bodied swimmers reached > 1.7–1.8 m s^−1^ at fast speeds, which make emerge superposition pattern of arm coordination^25,26^ and potential higher arm coordination asymmetry^4^ than at slow speeds.

In conclusion, contrary to able-bodied swimmers, most of the Para swimmers with unilateral physical impairment or visual impairment exhibited motor coordination asymmetry whatever the breathing conditions and the swimming paces. Our findings showed high probability for unilateral physical impairment and unilateral breathing preference to occur on the same side and mainly predicated by arm coordination asymmetry on the same side, suggesting the joined effect of unilateral impairment and unilateral breathing. Visual impairment and unilateral breathing preference can also jointly impact motor coordination symmetry as part of these Para swimmers exhibited asymmetric motor coordination. Practical implications for the coaches could be to firstly assess potential asymmetry of arm coordination and related effect of impairment and unilateral breathing before advising Para swimmers for a preferred breathing pattern. Last, motor asymmetries in Para swimmers should be considered as functional adaptations to organismic constraints (i.e. physical and visual impairment) instead of a mistake because of the comparison to able-bodied swimmers.

## Methods

### Participants

Inclusion criteria involved only (1) elite Para swimmers (2) with *unilateral breathing* preference (i.e. breathing laterality), and (3) with physical and visual impairment, which associated to unilateral breathing action could lead to asymmetric motor coordination. In particular, Para swimmers with unilateral physical impairment (e.g. amputation, agenesis, etc.) from classes S8, S9 and S10, and with visual impairment from classes S11, S12 and S13 were eligible. Therefore, our sample was composed of one elite Para triathlete and 14 elite Para swimmers. The Para triathlete is six-time World Para triathlon Champion and two-time Paralympic triathlon Champion; 7 Para swimmers are Paralympic or international Para swimmers referenced in the World Para Swimming ranking (https://www.paralympic.org/swimming/rankings) including one European Champion of the 100 m front crawl and 7 Para swimmers of the national team. Among this sample, 10 participants have unilateral physical impairment (2 with upper-limb impairment and 8 with lower-limb impairment, classified as S8, S9 or S10 and PTS4) and 5 have visual impairment (from classes S11, S12 or S13) as defined by the International Paralympic Committee (https://www.paralympic.org/classification). They all had a unilateral breathing preference. The sample included seven females of age 20.1 ± 2.3 years, height 159.4 ± 7.2 cm, mass 49.5 ± 5.6 kg and eight males of age 22.9 ± 6.7 years, height 179.6 ± 7.1 cm, mass 67.1 ± 7.7 kg (see other characteristics in Table 5).

**Table 5:** Characteristics of the paraswimmers.

Swimmer ID	Gender	Class	Impairment	Breathing laterality
1	Male	S9	Double clubfoot with higher impairment on right side	Right
2	Female	S9	Right lower leg agenesis	Right
3	Female	S10	Right lower arm agenesis	Left
4	Female	S8	Right arm agenesis	Left
5	Male	S10	Double clubfoot with higher impairment on left side	Left
6	Male	S10	Left lower leg amputation	Left
7	Female	S10	Left lower limb disease	Right
8	Male	S10	Left hand agenesis	Right
9	Female	S10	Left lower leg disease	Right
10	Male	PTS4	Right lower leg amputation	Right
11	Male	S11	Visual impairment	Right
12	Male	S13	Visual impairment	Left
13	Male	S13	Visual impairment	Left
14	Female	S12	Visual impairment	Right
15	Female	S11	Visual impairment	Left

All participants had at least 6 years of experience in competitive swimming programs, with at least 10 h of training per week. The participants voluntary participated in this study and provided written informed consent. All procedures were performed according to the Declaration of Helsinki, approved by the National Ethics Committee (national agreement number: 2021-A01186-35) and have been registered in http://www.clinicaltrials.gov/ (NCT05011591).

### Protocol

Participants performed their warm-up routine in the water during at least 20 min. Thereafter, the Para swimmers were required to perform 8 times 25-m composed of four breathing conditions (breathing every three strokes, apnea, breathing every two strokes on preferred breathing side, and on non-preferred breathing side) × two swimming paces (slow pace i.e. 400 m pace and fast pace i.e. 50 m pace) in a randomized order. Each trial started in-water with an underwater glide that should not exceed 5 m. Swimming speed was self-paced and a speed increase of minimum 40% between the slowest and the fastest paces was requested to validate the trial; finally, a 3 min rest was allowed between each trial (adapted from the previous study of Seifert et al.^8^).

### Data collection

The Para swimmers were equipped during the whole test with five inertial measurement units (IMUs) (Wavetrack Inertial System, Cometa, Milan, Italy) to assess arm and leg kinematics. Two IMUs were positioned on the dorsal side of the right and left forearms about 10 cm above the styloid apophysis of the radius, two IMUs were positioned on the medial surface of each tibia, about 10 cm above the medial malleolus, and one IMU was positioned on the sacrum. For the three Para swimmers with lower limb amputation, the IMUs were positioned on the anterior side of the thigh, just above the knee. The other Para swimmers did not request any adaptation of the IMU positioning. X-axes of each IMU were pointing up, i.e. aligned to the gravity axis; while Y-axes of each IMU were aligned with the medio-lateral axis of the body. Each IMU was waterproofed, weighted 10 g, measured 33 × 25 × 7 mm (length, width, height), and was composed of a three-dimensional accelerometer (maximum range ± 16 g), a three-dimensional gyroscope (maximum range ± 2000°/s) and a three-dimensional magnetometer (range of ± 4800µT). Each IMU registered as an individual data logger with a sampling frequency of 2000 Hz. Several remote controllers, held outside the water, started, and stopped the recordings to automatically synchronize all IMUs (one remote controller per participant) before asking the Para swimmers to enter the water. A unique recording was performed to collect the data continuously during the whole protocol. The IMUs were fixed to the skin with a therapeutic strap (Tegaderm Roll 16004S, 3 M) and adhesive tape (Leukotape K, 76,168–00, BSN Medical) similarly to Guignard et al.^27^.

Moreover, each trial was also recorded by a multi-cameras system to obtain swimming speed (S, in m s^−1^) and stroke length (SL, in m.cycle^−1^) for each cycle: Athletes In Motion (AIM) system (AIMSys Sweden AB, Lund, Sweden) (for more details, see^28,29^). In the present study, the system consisted of 22 stationary digital video cameras. Ten cameras were mounted above water (3 m high), and 12 were mounted beneath the water surface (behind windows) along with the 50 m pool. Each camera was 5 m apart, except the first and the last cameras both above and underwater that were positioned 2.5 m away from the start and end walls of the swimming pool. Two underwater cameras were positioned longitudinally from the swimmer displacement, at the centre of the water line. All cameras were placed perpendicular to the swimming direction 0.70 m beneath the water surface. The distance from the underwater cameras on the side to the water line was 5.5 m. The cameras were Axis Q3505-VE Mk II Network Camera (Axis AB, Lund, Sweden) above water and Axis Q1635 Network Camera (Axis AB, Lund, Sweden) underwater. The sampling frequency was 50 Hz, and the camera resolution was 1080 pixel per inch. It induces an increment of 0.02 s between two consecutive frames of the video: whenever a point under interest was observed (e.g., water hand entry), the operator noted the clock value. The synchronization between cameras and IMUs systems were performed by rapid and dynamic strikes on the IMUs positioned on the left forearm, in front of the cameras filming the swimmer from a sagittal point of view.

### Data analysis

To avoid start and finish effects and keep only the clean swimming parts of the lap, only the 5 to 7 cycles between 10 and 20 m have been used for data processing. Swimming speed was assessed as the time difference between the centre of the head passing the 10 and the 20 m lanes based on AIM system recording. The stroke length was also computed from AIM system for each cycle (time difference between two consecutive entries of the same hand in water) and then averaged over the cycles taken between 10 and 20 m. The arm and leg kinematics have been computed from the IMUs using MATLAB R2020b (The MathWorks, Inc. Natick, MA, USA).

### Arm coordination symmetry

The front crawl arm stroke cycle can be divided into four phases (i.e., catch and glide, pull, push and recovery), for which the relative duration was expressed in % of one arm stroke cycle duration^30^. The catch and glide and recovery are identified as non-propulsive phases while the pull and push are identified as propulsive phases. The full procedure to detect the beginning of pull, push and recovery phases using accelerometric and gyrometric data was extensively previously described^31,32^. The medial–lateral angular velocity of the forearm was filtered with Butterworth low-pass filter with cut-off frequency of 20 Hz and was used to detect the entry of the hand in water^32^. This corresponds to the first observable peak on the raw gyroscopic data between the instant of maximum angular velocity (corresponding to the half arm recovery) and the start of the pull. From there, the duration of one arm stroke cycle (T, in s), corresponding to the absolute time separating one hand water entry to the next entry of the same hand, has been computed and then the arm stroke rate (SR, in Hz) was obtained as follows (Eq. 1):

1 $${\text{SR }} = {1}/{\text{T}}$$

Finally, absolute stroke cycle durations were time-normalized (i.e., one complete stroke cycle is 100%) to allow averaging between cycles.

The arm coordination was assessed by the index of coordination (IdC) which quantifies the time gap between the propulsive phases of the two arms^30^. IdC was computed as the mean of IdC_left_ (Eq. 2) and IdC_right_ (Eq. 3):

2 $${\text{IdC}}_{{{\text{left}}}} = \left( {\left( {{\text{Time}}_{{\text{End of propulsive phase for left arm at cycle 1}}} {-}{\text{Time}}_{{\text{Beginning of propulsive phase for right arm at cycle 1}}} } \right) \cdot {1}00} \right)/{\text{T}}$$

3 $${\text{IdC}}_{{{\text{right}}}} = \left( {\left( {{\text{Time}}_{{\text{End of propulsive phase right arm at cycle 2}}} {-}{\text{Time}}_{{\text{Beginning of propulsive phase for left arm at cycle 1}}} } \right) \cdot {1}00} \right)/{\text{T}}$$

The IdC is expressed in percentage of the cycle duration: IdC > 0% corresponds to superposition pattern, IdC < 0% corresponds to catch-up pattern and IdC = 0% corresponds to opposition pattern^30^.

To account for arm coordination symmetry, IdC_left_ and IdC_right_ were normalized to systematically become positive by adding a high constant value (30%) (as previously done by Seifert et al.^8^). According to Seifert et al.^8^ and Herzog et al.^33^, the symmetry index (SI_IdC_) was computed as follows (Eq. 4):

4 $${\text{SI}}_{{{\text{IdC}}}} = (({\text{IdC}}_{{{\text{right}}}} {-}{\text{IdC}}_{{{\text{left}}}} )/0.{5} \cdot ({\text{IdC}}_{{{\text{right}}}} + {\text{IdC}}_{{{\text{left}}}} )) \cdot {1}00$$

where − 10% < SI_IdC_ < 10% indicates symmetry between right and left sides, SI_IdC_ < − 10% indicated asymmetry to the right side (i.e. smaller value on the right side than on the left side), and SI_IdC_ > 10% indicated asymmetry to the left side (i.e. higher value on the right side than on the left side). Then, the absolute value of SI_IdC_ was used to distinguish asymmetry (SI_IdC_ > 10%) from symmetry (10% > SI_IdC_ > 0%) to perform the statistical analysis.

### Leg coordination symmetry

The medial–lateral angular velocity of each IMU positioned on the distal portion of the leg was filtered with Butterworth low-pass filter with cut-off frequency of 10 Hz. Then, the maximal and minimal angular velocities were detected in order to estimate the downward and upward leg kicking duration, and finally the duration of one leg kicking cycle (Kicking Time or KT, in s) (according to Fulton et al.^34^). In other words, the KT corresponds to the absolute time separating the beginning of the downward leg kicking at cycle 1 to beginning of the downward leg kicking at cycle 2; then the leg kicking rate (KR, in Hz) was obtained for the left leg (KR_left_) and the right leg (KR_right_) (Eq. 5):

5 $${\text{KR }} = { 1 }/{\text{ KT}}$$

Then, according to Herzog et al.^33^, the symmetry of the left–right leg kicking rate (SI_KR_) was computed as follows (Eq. 6):

6 $${\text{SI}}_{{{\text{KR}}}} = (({\text{KR}}_{{{\text{right}}}} {-}{\text{KR}}_{{{\text{left}}}} )/0.{5} \cdot ({\text{KR}}_{{{\text{right}}}} + {\text{KR}}_{{{\text{left}}}} )) \cdot {1}00$$

where − 10% < SI_KR_ < 10% indicates symmetry between right and left sides, SI_KR_ < − 10% indicated asymmetry to the right side (i.e. smaller value on the right side than on the left side), and SI_KR_ > 10% indicated asymmetry to the left side (i.e. higher value on the right side than on the left side) (according to Herzog et al.^33^). Then, the absolute values of SI_KR_ were used to distinguish asymmetry (SI_KR_ > 10%) from symmetry (10% > SI_KR_ > 0%) to perform the statistical analysis.

### Statistical analysis

First, the effect of breathing conditions and swimming paces on the stroking parameters (i.e. SL, SR, KR and S) and on the motor coordination (i.e. IdC, the absolute value of SI_IdC_, the absolute value of SI_KR_) have been tested by two-way repeated measures ANOVAs (within-factor: breathing condition, swimming pace; covariate: swimmers). Sphericity in the repeated measures design was verified with the Mauchly test^35^. When the assumption of sphericity was not met, the significance levels of *F*-ratios were adjusted according to the Greenhouse–Geisser procedure^36^. Post-hoc pairwise conditions comparison tests were applied and family-wise error rate was controlled by applying a Bonferroni correction of the *p*-value^37^. Partial eta squared (*η*_P_^2^) statistics were calculated as an indicator of effect size, considering that *η*_P_^2^ = 0.01 represents a small effect, *η*_P_^2^ = 0.06 represents a medium effect and *η*_P_^2^ = 0.15 represents a large effect^38^.

Second, the relationships between the impairment side, the breathing side and the motor coordination symmetry have been examined and modelled by multinomial logistic regression. Multinomial logistic regression is used for categorical variables, and for which there are more than two categories. In our study, physical impairment and breathing laterality could be located either on the right or on the left side, and can be combined in various categories (e.g. a Para swimmer might have left arm amputation but left breathing preference, while another Para swimmer could have left arm agenesis and left breathing preference). Therefore, multinomial logistic regression has been used to predict the probability of the 6 categories to occur as regard of the observed values of SI_IdC_ and SI_KR_. The 6 categories have been built as follows: right breathing laterality & right impairment, left breathing laterality & left impairment, right breathing laterality & left impairment, left breathing laterality & right impairment, right breathing laterality & visual impairment, left breathing laterality & visual impairment. The model fitting was assessed through the Akaike Index Criterion (AIC) and the Nagelkerke regression coefficient (*R*^2^_N_). The model coefficients were based on the odds ratio, the estimate log odds ratio and the estimate marginal means^39^. The criteria for including in the model the covariable predictors was the omnibus Likelihood ratio tests^40^. All tests were performed using JAMOVI software (version 2.3.28, 2022, https://www.jamovi.org), with a level of statistical significance fixed at *p* < 0.05.

## Data Availability

The datasets generated during and/or analysed during the current study are available from the corresponding author on reasonable request.
